# Assessment of the psychometric properties of the eHealth Literacy Scale for Brazilian adolescents

**DOI:** 10.1371/journal.pone.0314099

**Published:** 2024-11-25

**Authors:** Mariane Carolina Faria Barbosa, Ana Luiza Peres Baldiotti, Júlia Lara Resende, Matheus de França Perazzo, Ramon Targino Firmino, Ana Flávia Granville-Garcia, Saul Martins Paiva, Fernanda de Morais Ferreira

**Affiliations:** 1 Department of Pediatric Dentistry, Federal University of Minas Gerais (UFMG), Belo Horizonte, (MG), Brazil; 2 Postgraduate Program in Dentistry, Federal University of Goiás (UFG), Goiânia, (GO), Brazil; 3 Postgraduate Program in Dentistry, Federal University of Campina Grande (UFCG), Campina Grande, (PB), Brazil; 4 Postgraduate Program in Dentistry, State University of Paraíba (UEPB), Campina Grande, (PB), Brazil; Federal University of Rio de Janeiro: Universidade Federal do Rio de Janeiro, BRAZIL

## Abstract

With the increase in digitalization of adolescents and health services, the population must demonstrate digital health literacy skills to be able to navigate online health information, as well as identify, evaluate, and apply relevant information. The present study aimed to investigate the psychometric properties of the adapted version of the eHealth Literacy Scale (eHEALS-BrA) for Brazilian adolescents. This study was conducted between September 2022 and May 2023, involving a total of 260 adolescents aged 13 to 19, with a mean age of 15.64 years (SD = 1.84), all of whom were enrolled in public schools in Brazil. Structural validity was evaluated by confirmatory factor analysis (CFA). The reliability of the instrument was assessed using Cronbach’s alpha (α) and McDonald’s omega (ω), and test-retest reliability. Questionnaires about sociodemographic aspects, health-related characteristics, and internet use were administered and used as discriminant validation measures. Convergent Validity was determined by correction with the domains of the World Health Organization Quality of Life-Bref (WHOQOL-Bref) instrument. For test-retest reliability, 67 participants completed the eHEALS-BrA a fortnight later. The average eHEALS-BrA score was 28.05 points (SD = 5.05). CFA revealed that the model with the best fit had a single factor (χ^2^ = 49.884 [df = 20, p = 0.0002], CFI = 0.934, TLI = 0.908, RMSEA = 0.076(CI:0.05–0.10), and SRMR = 0.045). The instrument demonstrated good reliability, with satisfactory internal consistency (α and ω = 0.71) and stability (ICC = 0.82, 95% CI:0.70–0.89). The eHEALS-BrA was correlated with the physical, psychological, and environmental domains of the WHOQOL-Bref (p = 0.001) (convergent validity). Higher scores were found among male adolescents, individuals who practiced physical activity, those with better self-rated health, those who considered the internet useful for searching for health information (discriminant validity), and those with better self-rated skills related to using the internet (convergent validity) (p < 0.05). The eHEALS-BrA demonstrated adequate psychometric properties for measuring digital health literacy in Brazilian adolescents.

## Introduction

Adolescence is the transition period between childhood and adulthood and is considered a stage of development in which important physical, neural, cognitive, behavioral, and psychological maturation occurs [1, 2]. Adolescents experience an increase in awareness about the body and health, a reduction in parental influence and a greater need for acceptance by peers [2, 3].

Adolescents are digital natives, that is, a generation that was born in the era of the popularization of the internet and that is growing up in a society that uses computers, tablets, and smartphones on a daily basis [2, 4]. Thus, social media are indispensable for the lives of these young people, who use digital platforms for entertainment purposes, searching for information, and communication [2, 5–7]. Moreover, adolescents often use the internet to better understand and manage their health. Indeed, the internet has become an attractive interactive resource with easy access to a wide range of health content and allows anonymous searches for sensitive information, such as that related to sexual and mental health [6, 8]. In Brazil, an estimated 90.2% of adolescents are connected to the internet and use this resource to obtain information [9, 10]. However, it is important to note that being born and raised in a highly digitalized environment does not necessarily mean they possess the skills to use it effectively [11].

The availability of high-quality health information in social media can significantly impact an individual’s health outcomes [6]. Nevertheless, information on the internet comes from a wide variety of servers and has sources that are difficult to control, which can lead to problems related to the quality of the information posted and the risk of biased content circulating in the media [12–14]. As adolescents could be highly sensitive to acceptance and are developing reflective processes and cognitive control, these young people may be vulnerable to misinformation and may have difficulty identifying reliable content [2, 6, 15].

Digital health literacy (DHL) can be defined as the ability of individuals to search, find, understand, and health information posted online and apply knowledge assertively to address or solve a health problem [16]. With this aspect in mind, Norman and Skinner developed the eHealth Literacy Scale (eHEALS) [17], a widely used self-report scale composed of eight items that measure DHL in a wide range of age groups and populations [18–22]. This instrument has recently been validated for adults [23] (18 to 80 years old) and university students [24] in Brazil.

Considering the peculiarities of adolescence and the importance of measuring DHL in this age group, eHEALS was recently cross-culturally adapted for adolescents in Brazil [25]. For this adaptation, the following steps were carried out: a) assessment and adequacy of cultural equivalence by a committee of experts; b) back-translation; c) synthesis of back-translations; and d) cognitive testing with 42 Brazilian adolescents, using cognitive interviews with probing questions. However, for recommendation and utilization, it is necessary to conduct a psychometric evaluation of the adapted instrument with larger samples of Brazilian adolescents, including an analysis of its dimensional structure through test-retests and assessment of construct validity, using convergent and divergent validity [25]. Therefore, the aim of the present study was to assess the psychometric properties of the adapted version of the eHealth Literacy Scale (eHEALS-BrA) for Brazilian adolescents.

## Methods

### Sample

This methodological study assessed the psychometric properties of the eHealth Literacy Scale, which was previously adapted for Brazilian adolescents (eHEALS-BrA) [25]. The sample for this study comprised adolescents aged 13 to 19 who were enrolled in five public schools conveniently selected in Belo Horizonte, Minas Gerais, Brazil. The sample size was based on the recommendation of Anthoine et al. [26]: two to 20 individuals for each item on the instrument and an absolute minimum of 100 to 250 individuals. Thus, we determined a minimum sample of 160 participants (20 per item). For greater representativeness, schools distributed throughout the municipality were selected randomly and had different basic education development indices (IDEB-2019), an indicator of the quality of teaching at Brazilian public schools.

Adolescents of both sexes, native speakers of Brazilian Portuguese with access to the internet, and who were present on the data collection days were included. Adolescents up to 12 years of age were excluded due to the 1998 Children’s Online Privacy Protection Act, which determines a minimum age of 13 years for creating accounts on social media and using digital services. Adolescents who were illiterate and those who had self-reported or school-reported problems (vision, hearing, or cognitive) that made it impossible to participate in the study were also excluded.

### Instrument

The eHealth Literacy Scale is a self-report instrument for measuring digital health literacy [17] developed in Canada’s English language and cultural context. The scale consists of eight items and the response options for each item are organized on a five-point Likert scale ranging from “strongly disagree” to “strongly agree”, depending on the respondent’s perception. The total score ranges from 8 to 40 points, with higher scores denoting a better self-perception of digital health literacy [17]. Despite having been validated for Brazilian adults [23] and university students [24], the version for adolescents in Brazil (eHEALS-BrA) [25] has not previously had its psychometric properties assessed. The full version of the eHEALS in Brazilian Portuguese adapted for adolescents is available in the S1 File of this manuscript.

### Data collection

This study was conducted from August to December 2022. Guardians first answered a questionnaire on their sociodemographic characteristics (age, degree of kinship to the adolescent, education, and income) and those of the adolescent (birth order, change in health, and use of medications). The adolescents answered the following instruments: the eHEALS-BrA (digital health literacy) [25], the World Health Organization Quality of Life-Bref (WHOQOL-Bref) [27, 28], and a questionnaire addressing their demographic characteristics (sex, birth order, age, education, relationship to guardian, guardian’s schooling), general health (regular physical activity, self-assessment of general and oral health), internet access (place of access, main means of access, mobile internet, frequency of access, social network accounts), self-assessment of skills related to using the internet, and search behavior for health information (researched a doctor/dentist, followed bloggers’ recommendations, uses the internet to read/search for information on health, schedule appointments, use health apps, research symptoms, and ask questions to a health professional).

Additionally, we used two introductory questions from the original eHEALS instrument: “How useful do you think the internet is in helping you make decisions about your health?” (not at all useful, not very useful, not sure, useful, very useful) and “How important is it for you to be able to access health information/resources available on the internet?” (not important, not very important, not sure, important, very important) [17].

The WHOQOL-Bref is an abbreviated version of the World Health Organization Quality of Life questionnaire (WHOQOL-100) consisting of 26 questions with response options on a Likert scale (1 to 5) [27, 28].

To evaluate stability, the eHEALS-BrA was re-administered to 25% of the sample within a 15-day interval following the initial application. Participants were selected to ensure that representation from each school was maintained. As in the first application, they completed the printed version of the instrument in a self-administered format in a designated room at their school.

### Statistical analysis

Data were analyzed using SPSS Statistics, version 21.0 (IBM Corp., Armonk, NY, USA) and Mplus software (Muthén & Muthén, version 8.2, Los Angeles, CA, USA). Confirmatory factor analysis (CFA) was performed to test the dimensionality of the instrument based on theoretical knowledge and empirical research [29]. One-, two-, and three-factor hypotheses [17, 21–24] were tested. In AFC the analytic approach used estimation adjusted using the mean- and variance-adjusted weighted least squares. Different statistical parameters were employed to assess the goodness of fit of the model, such as the Comparative Fit Index (CFI), Tucker-Lewis index (TLI), root mean square error of approximation (RMSEA), and standardized root mean square residual (SRMR). For the CFI and TLI, values ≥ 0.95 were considered indicative of an excellent fit and values between 0.90 and 0.95 were considered acceptable. RMSEA values ≤ 0.06 were considered indicative of an excellent fit of the model and values between 0.06 and 0.08 were considered acceptable [30, 31]. For the SRMR, values < 0.08 were considered indicative of a good fit [20, 21]. Communalities and factor loadings equal ≥ 0.4 were considered acceptable.

Reliability was investigated using two measures of internal consistency: Cronbach’s alpha (α) and McDonald’s omega (ω). For both coefficients, values ≥ 0.70 were considered adequate [32, 33]. Instrument stability and test-retest reliability were measured using the intraclass correlation coefficient (ICC), which was interpreted as follows: ≤ 0.40 = weak correlation; 0.41–0.60 = moderate correlation; 0.61–0.80 = good correlation; 0.81–1.00 = excellent correlation [30].

Construct validity was measured based on convergent and discriminant validity considering the eHEALS validation method and theoretical framework. The Mann-Whitney and Kruskal-Wallis U tests were used to identify the convergent and discriminant validity of the eHEALS-BrA by comparing the total score between categories of demographic variables, self-perception of general and oral health, internet access, and search behavior for health information on social media platforms. Spearman’s correlation coefficients were calculated to demonstrate the convergent validity of the eHEALS-BrA in relation to the WHOQOL-Bref.

### Ethical aspects

This study was conducted with authorization from the authors of the original instrument [17], received approval from the Human Research Ethics Committee of the Federal University of Minas Gerais (certificate number: 58603022.8.0000.5149), and followed the ethical principles stipulated in Resolution 466/2012 of the National Board Health Council and the Declaration of Helsinki.

## Results

The validity and reliability of the eHEALS-BrA were determined in a sample of 260 adolescents with a mean age of 15.63 years (SD: 1.84), 142 of whom (54.6%) were girls and 139 (53.7%) had up to eight years of schooling. Average age of the guardians was 43.35 years (± 8.14), with guardian’s education ≥ nine years of study (79.1%) and an average family income of R$2780.69 (US$526.64).

According to the CFA analysis, the model with the best fit was composed of a single factor. The goodness-of-fit statistics were χ^2^ = 49.884 (DF = 20, p = 0.0002), CFI = 0.934, TLI = 0.908, RMSEA = 0.076 (0.050–0.102) and SRMR = 0.045, indicating a good fit of the model. Factor loadings for the single-factor solution ranged between Q1 = 0.519 and Q4 = 0.656 (Table 1).

**Table 1:** Factor loadings for sigle-factor solution of eHEALS-BrA.

Item	Single- factor
**Q1**	0.519
**Q2**	0.581
**Q3**	0.544
**Q4**	0.656
**Q5**	0.595
**Q6**	0.556
**Q7**	0.586
**Q8**	0.575

The mean eHEALS-BrA score was 28.05 (SD = 5.05; range: 12 to 40) and mean administration time was 3.06 minutes. The instrument demonstrated good reliability. Internal consistency of the total scale was adequate (Cronbach’s alpha = 0.71 and McDonald’s omega = 0.71). Table 2 displays details on the means, variances, and Cronbach’s alpha if an item were deleted from eHEALS-BrA as well as item-total correlations and communalities. Test-retest reliability analysis demonstrated excellent reproducibility [ICC = 0.818 (95% CI: 0.70 to 0.89, p < 0.001)].

**Table 2:** Communalities, scale means, scale variances, item-total correlations and Cronbach’s alpha if item deleted from eHEALS-BrA.

Item	Scale mean if item deleted	Scale variance if item deleted	Item-total correlation	Cronbach’s alpha if item were deleted	1-factor communalities
**Q1**	24.1	21.7	0.35	0.69	0.27
**Q2**	24.2	20.1	0.40	0.68	0.34
**Q3**	24.8	21.2	0.39	0.68	0.30
**Q4**	24.4	20.3	0.47	0.66	0.43
**Q5**	24.0	20.6	0.41	0.68	0.35
**Q6**	24.8	19.2	0.39	0.68	0.31
**Q7**	24.5	18.9	0.42	0.68	0.34
**Q8**	25.3	19.7	0.39	0.68	0.33

Table 3 displays the mean eHEALS-BrA score according to demographic characteristics, health behaviors, and self-perception of general and oral health. Discriminant validity of the instrument was demonstrated by higher scores among male adolescents (p = 0.037), adolescents with better self-rated general health (p = 0.007), and those who performed physical activity regularly (p < 0.001). Table 4 displays the average eHEALS-BrA score according to access to social media and search behaviors for health information on the internet. Higher eHEALS-BrA scores were found among adolescents who considered the internet very useful/useful for making health decisions (p < 0.001) and those who considered it very important/important to be able to access health information and/or resources on the internet (p = 0.036).

**Table 3:** Mean (±SD) eHEALS-BrA scores according to demographic characteristics, health behaviors, and self-perception of general and oral health.

Variable		N (%)	eHEALS-BrA	p
			Mean (±SD)	
Sex	Female	142 (54.6)	27.48 (5.2)	0.037
	Male	118 (45.4)	28.72 (4.7)	
Birth Order	Only child	43 (17.1)	28.83 (5.2)	0.514*
	Oldest child	87 (34.5)	28.26 (4.5)	
	Middle child	39 (15.5)	26.79 (6.1)	
	Youngest child	83 (32.9)	28.05 (4.7)	
Health change	Yes	88 (34.5)	28.09 (5.1)	0.967
	No	167 (65.5)	28.02 (5.0)	
Medication use	Yes	85 (33.5)	28.40 (4.8)	0.401
	No	169 (66.5)	27.86 (5.1)	
Kinship of guardian	Mother	204 (81.0)	27.85 (5.1)	0.257*
	Father	31 (12.3)	29.30 (4.6)	
	Other	17 (6.7)	28.15 (4.6)	
Guardian’s schooling	Up to 8 years	53 (20.9)	26.90 (5.0)	0.062
	≥ 9 years	201 (79.1)	28.34 (5.0)	
Adolescent’s schooling	Up to 8 years	139 (53.7)	28.25 (4.7)	0.854
	≥ 9 years	120 (46.3)	27.77 (5.4)	
Self-rated general health	Very Good	53 (20.9)	29.47 (4.9)	**0.007**
	Good	134 (53.0)	28.20 (5.0)	
	Fair	61 (24.1)	26.66 (5.0)	
	Poor/Very Poor	5 (2.0)	23.80 (4.8)	
Self-rated oral health	Very Good	62 (24.4)	28.85 (4.5)	0.582
	Good	144 (56.7)	27.80 (5.5)	
	Fair	44 (17.3)	27.50 (4.5)	
	Poor/Very Poor	4 (1.6)	27.25 (4.6)	
Regular physical Activity	Yes	199 (78.3)	28.62 (4.7)	0.001*
	No	55 (21.7)	25.53 (5.4)	

Kruskal Wallis test/Mann-Whitney U test*

Convergent validity of the eHEALS-BrA was demonstrated by the significant correlation with three domains of the WHOQOL-Bref: physical (r: 0.228, p < 0.001), psychological (r: 0.229, p < 0.001), and environmental (r: 0.298, p < 0.001). Convergent validity was also demonstrated by the higher eHEALS-BrA scores among adolescents with a ’very good’ self-assessment of their abilities to use the internet (p = 0.001), those who had previously researched (p = 0.003) and read (p = 0.006) health information on the internet, those who used health apps (p = 0.003), and those who asked health-related question to a professional online (p = 0.034).

**Table 4:** Mean (±SD) eHEALS-BrA score according to social media access and searching for health information on internet.

Variable		N (%)	eHEALS-BrA	p
			Mean (SD)	
Where internet is accessed	Home	122 (48.2)	27.88 (5.2)	0.967
	Home and school	85 (33.6)	28.00 (4.9)	
	Home, school, and other places	46 (18.2)	28.07 (4.9)	
Main means of access	Cell phone	221 (87.0)	28.02 (4.9)	0.960
	Computer	21 (8.3)	27.43 (6.5)	
	Notebook	9 (3.5)	27.56 (6.6)	
	Others: tablet/TV	3 (1.2)	28.67 (3.0)	
Frequency of Internet use	Every day	233 (91.7)	28.09 (5.0)	0.458
	Almost every day	18 (7.1)	26.94 (5.9)	
	Almost never	3 (1.2)	26.00 (3.6)	
Do you have mobile Internet?	Yes	138 (54.3)	28.49 (5.3)	0.087
	Sometimes	94 (37.0)	27.18 (4.7)	
	No	22 (8.7)	28.18 (4.9)	
Self-rated internet use skills	Very good	101 (39.6)	29.53 (4.7)	**0.001**
	Good	124 (48.6)	27.40 (4.7)	
	Poor	30 (11.8)	25.10 (6.1)	
Do you have social network accounts?	Yes	252 (99.2)	28.00 (5.1)	0.276*
	No	2 (0.8)	24.50 (4.9)	
Searched for a doctor/dentist	Yes	200 (78.4)	28.23 (5.1)	0.093*
	No	55 (21.6)	27.07 (4.9)	
Followed bloggers’ recommendations	Yes	131 (51.4)	28.44 (5.0)	0.190*
	No	124 (48.6)	27.49 (5.1)	
Considers internet useful for making health decisions	Very useful/ Useful	133 (52.2)	29.51 (4.5)	**0.001**
	Not sure / Not very useful	117 (45.9)	26.34 (5.2)	
	Not at all useful	5 (2.0)	25.40 (4.4)	
Considers it important to access health information on internet	Very Important/ Important	174 (68.8)	28.45 (5.1)	**0.036**
	Not sure / Not very important	69 (27.3)	27.19 (4.8)	
	Not at all important	10 (4.0)	24.70 (5.6)	
Searched for health information on Internet	Yes	233 (91.4)	28.38 (4.6)	0.003*
	No	22 (8.6)	23.68 (7.3)	
Scheduled appointment	Yes	139 (54.5)	28.36 (5.4)	0.093*
	No	116 (45.5)	27.52 (4.6)	
Read health information	Yes	183 (72.0)	28.54 (4.9)	0.006*
	No	71 (28.0)	26.52 (5.3)	
Used health apps	Yes	88 (34.5)	29.26 (4.8)	0.003*
	No	167 (65.5)	27.30 (5.1)	
Asked a professional online	Yes	131 (51.4)	28.53 (5.2)	**0.034**
	No	124 (48.6)	27.39 (4.8)	
Searched for symptoms online	Yes	206 (80.8)	28.29 (4.9)	0.051
	No	49 (19.2)	26.65 (5.5)	

Kruskal Wallis test/Mann-Whitney U test*

## Discussion

The use of an assessment measure in a different culture, population, or age group should only be recommended after a careful process of cross-cultural adaptation and the assessment of its psychometric properties [34, 35]. The present study demonstrated that the eHEALS-BrA has good properties for measuring digital health literacy in Brazilian adolescents. Furthermore, the instrument is simple and fast to apply (approximately three minutes) and requires minimal training from the professional.

The mean total eHEALS-BrA score is similar to that found for university students in Brazil (28.0) [24] and Turkish adolescents (27.52) [36]. However, the mean score was higher than that found for adults [23], which may be explained by the greater familiarity of adolescents with information and communication technologies, which can increase their self-confidence in using such technologies for health purposes [6]. Furthermore, as adolescents are in a period of physical, emotional, psychological, and cognitive maturation [2], they may present better self-assessment (eHEALS) than actual LDS skills. Recently, a study carried out with Australian adolescents highlighted the discrepancy between adolescents’ perceived and actual digital health literacy, measured for served practical search tasks and follow-up interviews [37].

Good correlations were found between the items and total score, indicating that no item should be removed from instrument [38]. As occurred in the analysis of the original instrument [17], CFA revealed that the single-factor model had the best fit, with factor loadings ranging from 0.519 to 0.656 for the eight items on the scale. The dimensionality of an instrument is a fundamental psychometric property to determine the number of factors necessary to characterize the construct adequately. The single-factor solution was supported in most validations of this instrument [24, 39–41].

The eHEALS-BrA exhibited adequate internal consistency demonstrating good reliability. These values are lower than those found in the validation study of the original instrument with Canadian adolescents and young adults (13–21 years of age) (α = 0.88) [17] and cross-cultural adaptations for adolescents in Serbia (α = 0.88) [42] as well as Brazilian adults (α and ω = 0.95) [23] and university students (α = 0.88) [24]. Nonetheless, assessment measures with α ≥ 0.70 are considered acceptable and recommended for use [32, 33].

Furthermore, although this study’s alpha value is not very high, it is important to highlight that instruments with a low number of questions, poor inter-item correlation, or heterogeneous constructs may interfere with the evaluation. Additionally, while Cronbach’s alpha is not the most appropriate measure of internal consistency for multidimensional scales, it is described in the literature as a reliable measure for unidimensional instruments, as is the case in this study [43]. Despite this, to confirm these findings, we also used Omega as a measure to evaluate internal consistency [33].

In terms of reproducibility, the eHEALS-BrA exhibited excellent test-retest stability. The original authors used Pearson’s correlation test between four administration times, during which smoking interventions were conducted [17]. Therefore, it is not possible to compare our results. However, the ICC in the present study was higher than that found in the validation study involving Brazilian university students with a similar interval between the test and retest (ICC = 0.71) [24].

As occurred in the validation study of the original instrument, the average digital health literacy score was higher in male adolescents [17]. Furthermore, the eHEALS-BrA score was associated with better outcomes in terms of quality of life and self-rated general health as well as regular physical activity among the adolescents. A higher eHEALS-BrA score was positively associated with a better self-perception of skills in using the internet, asking questions online to a professional, searching for and reading health information on the internet, and using health applications. In line with our results, a recent study with Serbian adolescents found that being male, reporting greater use of the internet to research health, using health applications, and performing better on the eHEALS were associated with a greater influence of online health information on health-related decision-making [44].

Although a randomly selected sample is preferable for validation studies, it is not necessary for analyzing the psychometric properties of an assessment measure. It’s important to have a sample that reflects and captures the range of the target population [45]. This study was conducted with adolescent students in public schools within the same municipality, where we randomly selected schools with varying assessments of teaching quality and distribution throughout the area municipality.

The eHEALS has some limitations, as it measures an individual’s perception of health information from electronic sources and does not encompass the practical assessment of skills in searching, finding, understanding, and assessing this information. Patient-reported outcome measures (PROMs) are questionnaires that collect subjective information directly from patients regarding specific or general conditions, contributing to clinical and functional outcomes by transforming unmeasurable subjective qualities into quantitative measures [46, 47]. In this research, we used two PROMS, eHEALS-BrA and WHOQOL-Bref. PROMs are essential for health investigations, as their completion prompts patients to reflect on their health and encourages them to raise healthcare issues.

The eHEALS scale has the limitation of not evaluating all aspects related to the current scenario of high digital interaction (Health 2.0). It is worth noting that while other instruments have been developed to measure DHL, eHEALS is the most widely used to investigate this construct due to its pioneering nature, versatility for different age groups and cultural contexts, evidence of adequate psychometric properties, and ease of application [41]. Furthermore, in this study, we used related constructs and variables to Digital Health Literacy to measure its convergent validity because, during the data collection period, we did not have another cross instrument- Culturally adapted and validated for measuring Digital Health Literacy among adolescents in Brazil to compare with eHEALS-BrA.

The eHEALS has some limitations, as it measures an individual’s perception of health information from electronic sources and does not encompass the practical assessment of skills in searching, finding, understanding, and assessing this information. Patient-reported outcome measures (PROMs) are essential for health investigations, as their completion prompts patients to reflect on their health and encourages them to raise healthcare issues. PROMs are questionnaires that collect subjective information directly from patients regarding specific or general conditions, contributing to clinical and functional outcomes by transforming unmeasurable subjective qualities into quantitative measures [46, 47]. In this research, we used two PROMS, eHEALS-BrA and WHOQOL-Bref. We utilized related constructs and variables to Digital Health Literacy to measure its convergent validity because, during the data collection period, we did not have another instrument that was cross-culturally adapted and validated for measuring Digital Health Literacy among adolescents in Brazil to compare with eHEALS-BrA.

Furthermore, the eHEALS scale has the limitation of not evaluating all aspects related to the current scenario of high digital interaction (Health 2.0) [41]. It is worth noting that while other instruments have been developed to measure DHL, eHEALS is the most widely used to investigate this construct due to its pioneering nature, versatility for different age groups and cultural contexts, evidence of adequate psychometric properties, and ease of application [41].

With the acceleration of the digitalization of health services as a result of the COVID-19 pandemic and the worsening of the infodemic, there was a need to measure digital health literacy to identify the current situation in the country, formulate measures to promote the improvement of DHL, and disseminate reliable information based on scientific evidence to combat misinformation [48, 49].

The adaptation of eHEALS for Brazilian adolescents was the first attempt to obtain an instrument for measuring DHL in this age group in Brazil [25]. However, it was necessary to provide evidence of its psychometric properties. This study stands out due to the emerging need to investigate digital health literacy in different age groups to understand the challenges and disparities in access to health information in social media and the use of digital health services by the population. Future research is needed to assess the DHL of adolescents in Brazil, investigate gender differences in digital health literacy, and explore the associated health outcomes. Furthermore, it is necessary to advance instruments in Brazilian Portuguese that measure HDL more broadly (Health 1.0 and 2.0).

## Conclusion

The eHEALS-BrA demonstrated adequate psychometric properties for measuring the digital health literacy of Brazilian adolescents.

## Supporting information

S1 File eHealth Literacy Scale. (PDF)
