# Supplementary material for: Assessment of the psychometric properties of the eHealth Literacy Scale for Brazilian adolescents
Source: PLoS One. 2024 Nov 25;19(11):e0314099. doi: 10.1371/journal.pone.0314099 (PMC11588242; doi:10.1371/journal.pone.0314099)
Supplement: S1 File — (PDF) [file pone.0314099.s001.pdf]

Adolescent's Identification Number: (DO NOT FILL THIS FIELD): _______________
YOUR FULL NAME: ____

For the questions in the chart below, I would like to know your opinion and experience in the use of the Internet to obtain information on health. **There are no right or wrong answers.** For each statement, mark the answer that best reflects your opinion and experience at the moment.

1. I know **how** to find useful health information/resources on the Internet.
a) I fully disagree
b) I disagree
c) I am not sure
d) I agree
e) I fully agree

2. I know **how to use** the Internet to answer my questions about health.
a) I fully disagree
b) I disagree
c) I am not sure
d) I agree
e) I fully agree

3. I know **what** health resources are available on the Internet.
a) I fully disagree
b) I disagree
c) I am not sure
d) I agree
e) I fully agree

4. I know **where** to find useful health information/resources on the Internet.
a) I fully disagree
b) I disagree
c) I am not sure
d) I agree
e) I fully agree

5. I know how to use **the health information** that I find on the internet to help me.
a) I fully disagree
b) I disagree
c) I am not sure
d) I agree
e) I fully agree

6. I have the skills I need to **evaluate** health information/resources that I find on the internet.
a) I fully disagree
b) I disagree
c) I am not sure
d) I agree
e) I fully agree

7. I can differentiate health information/resources that are of **high quality** from those that are of **low quality** on the internet.
a) I fully disagree
b) I disagree
c) I am not sure
d) I agree
e) I fully agree

8. I feel **confident** in using information from the Internet to make health decisions.
a) I fully disagree
b) I disagree
c) I am not sure
d) I agree
e) I fully agree

Thank you!

Nº de identificação do adolescente: (NÃO PREENCHER ESTE CAMPO): _______________
SEU NOME COMPLETO: ____

Para as questões abaixo, eu gostaria de saber sua opinião e sua experiência no uso da Internet para buscar informações sobre saúde. **Não existem respostas certas ou erradas.** Para cada afirmação, marque a resposta que melhor reflete sua opinião e sua experiência neste momento.

Considere **"Recursos de saúde"** como todas as formas de usar a internet relacionada a saúde.

1. Eu sei **como** encontrar informações/recursos úteis de saúde na Internet.
a) Discordo totalmente
b) Discordo em parte
c) Não tenho certeza
d) Concordo em parte
e) Concordo totalmente

2. Eu sei **como usar** a Internet para responder as minhas dúvidas sobre saúde.
a) Discordo totalmente
b) Discordo em parte
c) Não tenho certeza
d) Concordo em parte
e) Concordo totalmente

3. Eu sei **quais** os recursos de saúde que estão disponíveis na Internet.
a) Discordo totalmente
b) Discordo em parte
c) Não tenho certeza
d) Concordo em parte
e) Concordo totalmente

4. Eu sei **onde** encontrar informações/recursos úteis de saúde na Internet.
a) Discordo totalmente
b) Discordo em parte
c) Não tenho certeza
d) Concordo em parte
e) Concordo totalmente

5. Eu sei como usar **as informações de saúde** que encontro na internet para me ajudar.
a) Discordo totalmente
b) Discordo em parte
c) Não tenho certeza
d) Concordo em parte
e) Concordo totalmente

6. Eu **tenho as habilidades** necessárias para avaliar as informações/recursos de saúde que encontro na internet.
a) Discordo totalmente

b) Discordo em parte
c) Não tenho certeza
d) Concordo em parte
e) Concordo totalmente

7. Eu consigo diferenciar as informações/recursos de saúde de **alta qualidade** das de **baixa qualidade** na internet.
a) Discordo totalmente
b) Discordo em parte
c) Não tenho certeza
d) Concordo em parte
e) Concordo totalmente

8. Eu me sinto **confiante** em usar as informações da Internet para tomar decisões sobre saúde.

a) Discordo totalmente
b) Discordo em parte
c) Não tenho certeza
d) Concordo em parte
e) Concordo totalmente

OBRIGADA!
